# Symbioses of alvinocaridid shrimps from the South West Pacific: No chemosymbiotic diets but conserved gut microbiomes

**DOI:** 10.1111/1758-2229.13201

**Published:** 2023-09-26

**Authors:** Pierre Methou, Valérie Cueff‐Gauchard, Loïc N. Michel, Nicolas Gayet, Florence Pradillon, Marie‐Anne Cambon‐Bonavita

**Affiliations:** ^1^ X‐STAR Japan Agency for Marine‐Earth Science and Technology (JAMSTEC) Yokosuka Japan; ^2^ Univ Brest Ifremer, CNRS, Unité Biologie des Environnements Extrêmes marins Profonds Plouzané France; ^3^ Laboratory of Oceanology, Freshwater, and Oceanic Sciences Unit of reSearch (FOCUS) University of Liège Liège Belgium

## Abstract

*Rimicaris exoculata* shrimps from hydrothermal vent ecosystems are known to host dense epibiotic communities inside their enlarged heads and digestive systems. Conversely, other shrimps from the family, described as opportunistic feeders have received less attention. We examined the nutrition and bacterial communities colonising ‘head’ chambers and digestive systems of three other alvinocaridids—*Rimicaris variabilis*, *Nautilocaris saintlaurentae* and *Manuscaris* sp.—using a combination of electron microscopy, stable isotopes and sequencing approaches*.* Our observations inside ‘head’ cavities and on mouthparts showed only a really low coverage of bacterial epibionts. In addition, no clear correlation between isotopic ratios and relative abundance of epibionts on mouthparts could be established among shrimp individuals. Altogether, these results suggest that none of these alvinocaridids rely on chemosynthetic epibionts as their main source of nutrition. Our analyses also revealed a substantial presence of several *Firmicutes* and *Deferribacterota* lineages within the foreguts and midguts of these shrimps, which closest known lineages were systematically digestive symbionts associated with alvinocaridids, and more broadly for *Firmicutes* from digestive systems of other crustaceans from marine and terrestrial ecosystems. Overall, our study opens new perspectives not only about chemosynthetic symbioses of vent shrimps but more largely about digestive microbiomes with potential ancient and evolutionarily conserved bacterial partnerships among crustaceans.

## Graphical abstract

This work examines the nutrition and diversity or bacterial communities colonising ‘head’ chambers and digestive systems of shrimp endemic to hydrothermal vents of the South West Pacific*.* Our results suggest that, unlike relatives from the Atlantic Ocean, none of these shrimps rely on chemosynthetic epibionts as their main source of nutrition. On the other hand, vent shrimps from the South West Pacific exhibit conserved bacterial lineages within their digestive microbiomes similar to those of other vent shrimps and more largely, other crustaceans from marine and terrestrial ecosystems suggesting a potential ancient and evolutionarily conserved host‐symbiont partnerships among crustaceans.

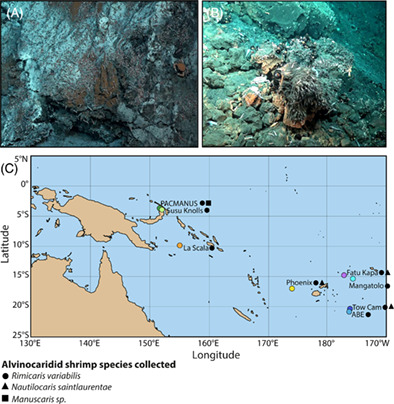

## INTRODUCTION

Microbial symbioses are a ubiquitous phenomenon in nature, expanding physiological capabilities and ecological niches of organisms (McFall‐Ngai et al., 2013). In many places, these associations constitute the structural base of ecosystems such as in hydrothermal vents. There, chemosynthetic symbioses with microorganisms using the chemical energy arising from vent fluid emissions are found in all invertebrates, establishing the foundation of lush faunal assemblages (Dubilier et al., 2008; Sogin et al., 2020).

Among them, *Rimicaris exoculata* and *Rimicaris kairei* form large aggregations of thousands of individuals, gathered in the close vicinity of fluid emissions respectively in the Mid Atlantic Ridge and the Central Indian Ridge (Zbinden & Cambon‐Bonavita, 2020). These shrimps host a complex community of cocci, rod‐shaped and filamentous epibionts on the inner side of their enlarged cephalothorax, that is, the branchiostegite, and on setae covering the surface of their hypertrophied mouthparts (Methou et al., 2022b; Petersen et al., 2010; Zbinden et al., 2004). These communities comprise a wide diversity of chemosynthetic partners including *Campylobacterota*, *α‐*, *γ‐* and *ζ‐Proteobacteria* as well as *Desulfobacterota* among others (Cambon‐Bonavita et al., 2021; Guri et al., 2012; Jan et al., 2014; Jiang et al., 2020; Methou et al., 2022b; Petersen et al., 2010; Zbinden et al., 2008), from which their hosts derive most of their nutrition (Gebruk et al., 2000; Methou et al., 2020; Polz et al., 1998; Van Dover, 2002) through direct transtegumental transfer of organic compounds (Ponsard et al., 2013). This diversity of bacterial partners reflects a diversity of metabolisms based on a wide range of energy sources (Cambon‐Bonavita et al., 2021; Jan et al., 2014; Jiang et al., 2020) enabling these animals to thrive in vent fields with contrasting profiles of fluid chemistries.

Besides, *R. exoculata* and *R. kairei* shrimps harbour another community of resident symbionts within their digestive system (Aubé et al., 2022; Durand et al., 2010, 2015; Guéganton et al., 2022; Qi et al., 2022; Zbinden & Cambon‐Bonavita, 2003). In *R. exoculata* this digestive symbiosis exhibits a clear partitioning between organs with several lineages of *Firmicutes* affiliated to *Mycoplasmatales* located in the foregut (oesophagus and stomach) and *Firmicutes* from the *Clostridia* class as well as *Candidatus* Rimicarispirillum which are long thin *Deferribacterota* inserted between microvilli in their midgut (Aubé et al., 2022; Guéganton et al., 2022). Unlike chemoautotrophic epibionts from the cephalothoracic cavity, these symbionts are heterotrophic and were hypothesised to complement their host diet and participate in their immunity (Aubé et al., 2022). To date, other bacterial lineages often found in the digestive microbiome of *R. exoculata* such as *Campylobacterota* and *Gammaproteobacteria* (Durand et al., 2010, 2015) were only observed as transient rod‐shaped and cocci cells in its alimentary bolus (Guéganton et al., 2022).

These symbiotic communities of the cephalothoracic cavity and the foregut are renewed alongside their host exoskeleton at each moult, whereas those from the midgut are maintained throughout their adult life (Corbari et al., 2008; Guri et al., 2012). The constant renewal of their microhabitat coupled with an absence of similar or closely related lineages in the surrounding environment of their host, question the transmission pathways of the Mycoplasmatales located in the foregut (Durand et al., 2015). Similarly, the lack of geographic clustering of *Deferribacterota* symbionts in the midgut of *R. exoculata*, which are also absent from the environment, suggests a maternal inheritance (Durand et al., 2015). However, these lineages were never detected on their egg broods during the entire embryonic development (Guri et al., 2012; Methou et al., 2019).

Apart from *R. exoculata* and *R. kairei*, symbioses have been found in two other alvinocaridid species, *R. hybisae* from the Mid Cayman Rise and *R. chacei* from the Mid‐Atlantic Ridge, which however display different trophic relations towards their symbiosis (Apremont et al., 2018; Assié, 2016; Nye et al., 2012). *R. chacei* shrimps lack an hypertrophied cephalothorax and are only partially dependent on their chemosynthetic symbiosis, with a mixed diet of symbiotrophy, bacterivory and scavenging (Gebruk et al., 2000; Methou et al., 2020). Their digestive system also hosts similar symbiotic communities than for *R. exoculata* with the same partitioning among foreguts and midguts (Apremont et al., 2018; Guéganton et al., 2022). On the other hand, *R. hybisae* shows more similarity with the ecology of *R. exoculata* and *R. kairei*, forming dense aggregates around chimneys and with an enlarged cephalothorax heavily colonised by epibionts (Nye et al., 2012; Streit et al., 2015). However, recent evidence from gut contents and isotopic compositions of *R. hybisae* individuals distributed at the vent site periphery suggest they might have retained an ability to feed on other sources, including facultative carnivory (Versteegh et al., 2022), in addition to chemosynthetic bacterial sources (Streit et al., 2015).

In other alvinocaridids, nutritional strategies have been hypothesised to be mostly opportunistic and scavenging, with the use of several food sources including bacterial mats, detritus or the predation of small invertebrates (Gebruk et al., 2000; Stevens et al., 2008; Suh et al., 2022; Van Audenhaege et al., 2019). Based on stable isotope compositions, it was suggested that *Rimicaris variabilis* and *Manuscaris* sp. from the Manus Basin could either be conventional grazers/scavengers or feed on episymbiotic autotrophic bacteria in a similar fashion to *R. exoculata* (Van Audenhaege et al., 2019). Yet, no extensive study has investigated the microbial communities from their branchiostegites, mouthparts or digestive system so far.

Our study explores the bacterial communities colonising cephalothoracic cavities and digestive systems of three alvinocaridid species from hydrothermal vents of South West Pacific basins—*Rimicaris variabilis*, *Nautilocaris saintlaurentae* and *Manuscaris* sp.—as well as their nutrition, using a combination of electron microscopy, multiple stable isotopes and sequencing approaches. Our aim was to examine symbiotic relationships across alvinocaridid species with distinct ecologies to better understand the role and evolution of these symbioses. We address the following questions: (1) Do alvinocaridid species described as opportunistic feeders host symbiotic communities in their cephalothoracic cavity and/or their digestive system? (2) Do these potential symbiotic communities comprise similar or related bacterial lineages to epibionts of other *Rimicaris* species? (3) Can these alvinocaridid species rely, at least partially, on chemosynthetic epibionts for their nutrition?

## EXPERIMENTAL PROCEDURES

### Field sampling

**FIGURE 1:**
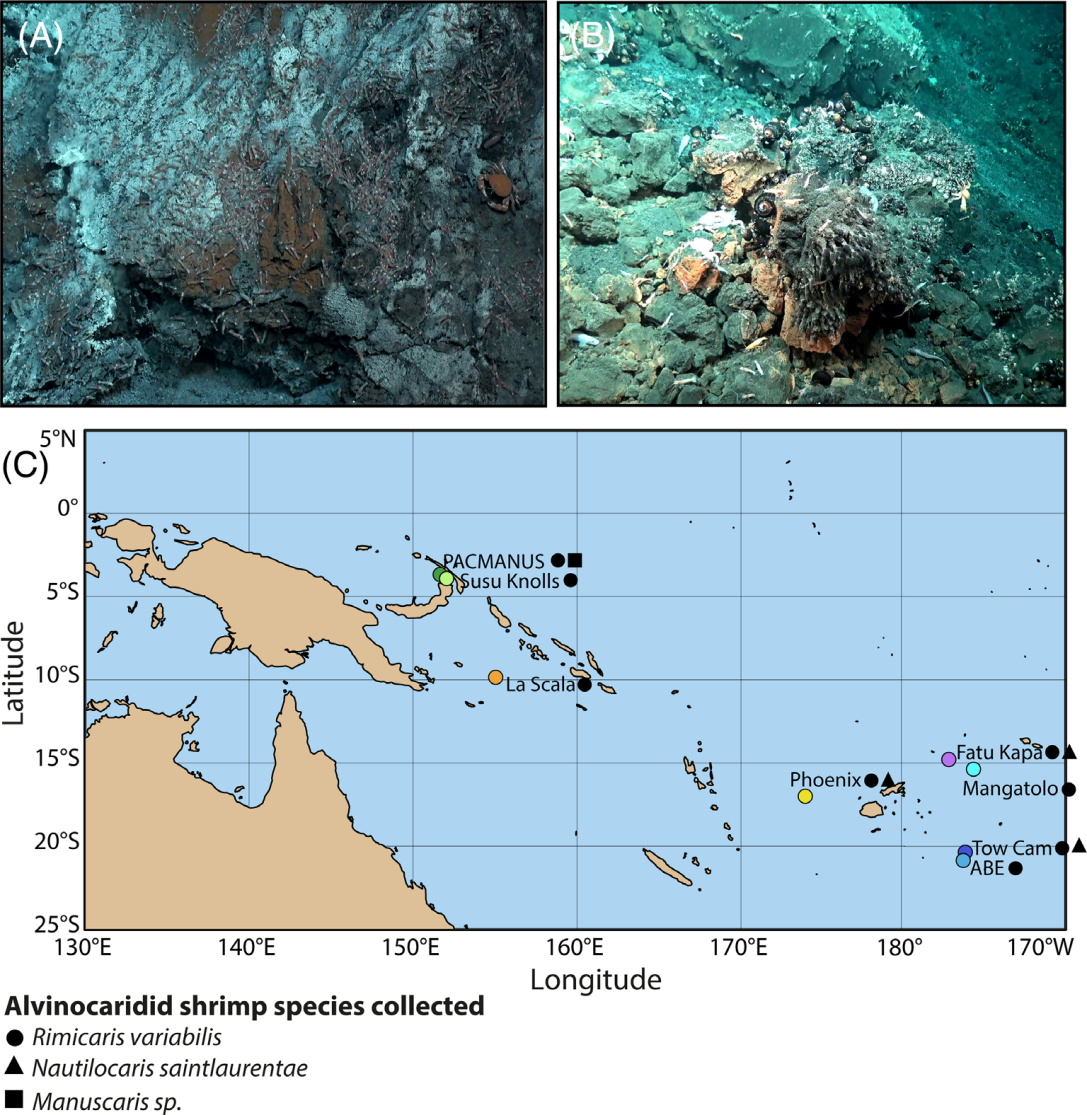
(A) Alvinocaridids shrimps on the wall of an active vent chimney at Pacmanus (Manus basin). (B) Alvinocaridids shrimps around assemblages of barnacles and at La Scala (Woodlark Basin). (C) Sampling localities of alvinocaridid shrimps from Southwest Pacific basins. Colour dots depict hydrothermal vent field locations. Shapes depict shrimp species collected at a given sampling field.

Alvinocaridid shrimps were collected during the Futuna3 2012 and CHUBACARC 2019 oceanographic expeditions on board the R/V *L'Atalante* using a suction sampler manipulated by the HOV Nautile and the ROV Victor 6000, respectively. A total of 81 *Rimicaris variabilis* individuals were sampled from eight hydrothermal vent fields: Pacmanus and Susu Knolls in the Manus basin, La Scala in the Woodlark basin, Phoenix in the North Fiji basin, Fatu Kapa in the Futuna volcanic arc and Mangatolo, ABE and Tow Cam in the Lau basin (Figure 1A–C). In addition, 25 *Nautilocaris saintlaurentae* individuals were also sampled at the Phoenix, Fatu Kapa and Tow Cam vent fields, as well as one *Manuscaris* sp. at the Pacmanus vent field. Specimens were identified morphologically and confirmed by genetic barcoding of their COI gene with specific primers for alvinocarids, using the protocol from (Methou et al., 2020). All sequences have been deposited in GenBank under accession numbers OQ363903—OQ364004 (see Table S1 for sampling summary with associated individual ID). The 16S rRNA dataset is available in the NCBI SRA repository (submission identifier SUB12697284 and BioProject identifier PRJNA932596).

Upon their recovery on board, dissected parts or whole shrimps were stored frozen at −80°C before processing for stable isotopes or 16S rRNA metabarcoding. Specimens were dissected directly on board or in a shore‐based laboratory under sterile conditions to retrieve their anatomical parts: scaphognathites and exopodites mouthparts as well as branchiostegites from the cephalothoracic cavity, foreguts and midguts of their digestive system and pieces of abdominal muscles. Pieces from cephalothoracic cavities and mouthparts were also fixed in a 2.5% glutaraldehyde filtrated seawater solution for 16 h at 4°C, rinsed and then stored at 4°C in filtrated seawater with 0.44 g/L of NaN_3_ at pH 7.4 until use for scanning electron microscopy (SEM) observations.

### Scanning electron microscopy

Dissected mouthparts and branchiostegites were dehydrated with an ethanol series (25%, 50%, 75% and 100% ethanol) and then for 5 h in a critical point dryer CPD 020 (Balzers Union, Balzers, Liechtenstein). Samples were then gold‐coated with an SCD 040 (Balzers Union). Observations and imaging were performed using a Quanta 200 microscope (FEI‐Thermo Fisher, Hillsboro, OR, USA).

### Stable isotope analysis

The abdominal muscle of alvinocaridid shrimps was oven‐dried to constant mass at 50°C (>48 h) and ground into a homogeneous powder using a mortar and pestle. Measurements of stable isotope ratio were performed by continuous flow–elemental analysis–isotope ratio mass spectrometry (CF‐EA‐IRMS) at University of Liège (Belgium), using a vario MICRO cube C‐N‐S elemental analyser (Elementar Analysensysteme GMBH, Hanau, Germany) coupled to an IsoPrime100 isotope ratio mass spectrometer (Isoprime, Cheadle, United Kingdom). Isotopic ratios were expressed in ‰ using the widespread δ notation (Coplen, 2011) relative to the international references: Vienna Pee Dee Belemnite (for carbon), atmospheric air (for nitrogen) and Vienna Canyon Diablo Troilite (for sulphur). Primary analytical standards used for these analyses were the following: Sucrose (IAEA‐C‐6; δ^13^C = −10.8 ± 0.5‰; mean ± SD), ammonium sulphate (IAEA‐N‐2; δ^15^N = 20.4 ± 0.1‰; mean ± SD) and silver sulphide (IAEA‐S‐2; δ^34^S = 22.6 ± 0.1‰; mean ± SD). A secondary analytical standard, Sulfanilic acid (Sigma‐Aldrich; δ^13^C = −25.6 ± 0.4‰; δ^15^N = −0.13 ± 0.4‰; δ^34^S = 5.9 ± 0.5‰; means ± SD) was also used as well as an internal laboratory standard (seabass muscle). These standards were analysed interspersed among samples with one replicate of each standard every 15 analyses. Standard deviations on multi‐batch replicate measurements of secondary and internal laboratory standards were 0.2‰ for δ^13^C and δ^15^N and 0.4‰ for δ^34^S.

SIBER (Stable Isotope Bayesian Ellipses in R; Jackson et al., 2011) was used to explore ecological niches in an R 4.2.1 statistical environment (R Core Team, 2020). Two separate sets of standard ellipses were constructed: one with δ^13^C and δ^15^N data and another with δ^13^C and δ^34^S data. Areas of these ellipses were also estimated using the Bayesian model (SEA_B_) with direct intergroup pairwise comparisons of SEA_B_. The model solutions were presented using credibility intervals of probability density function distributions. Areas of all ellipses were also estimated using the SEAc correction for small sample sizes, as outlined in (Jackson et al., 2011).

### DNA extraction and sequencing

Twenty‐three *Rimicaris variabilis*, six *Nautilocaris saintlaurentae* and one *Manuscaris* sp. specimens were used for DNA extraction of their mouthparts, foreguts and midguts, as well as 17 additional *R. variabilis* and two additional *N. saintlaurentae* for mouthparts only, using the Nucleospin® Soil Kit (Macherey‐Nagel, Germany) following manufacturer's instructions. Three blanks (i.e., a negative DNA extraction control) were also performed in parallel with DNA extractions of shrimp specimens.

For sequencing of the V3‐V4 variable region of 16S rRNA (Fadrosh et al., 2014) using Illumina's MiSeq technology, libraries were prepared using two successive PCR steps. (a) PCR1: samples were amplified in triplicate using the 341/785 primers (Herlemann et al., 2011) to generate a 450 bp fragment. The P5 and P7 Illumina primers were included in the 5′ part of the 341 forward and 785 reverse primers, with Truseq read 1 (CTTTCCCTACACGACGC TCTTCCGATCT) and Truseq read 2 Illumina adapters (GGAGTTCAGACGTGTGCTCTTCCGATCT) respectively. PCR1 amplifications were performed in a final volume of 50 μL using 1 or 2 μL of DNA, 1.25 U of TaqCore polymerase (MP Biomedicals), standard Buffer with final 1.5 mM MgCl2, 0.5 mM of each dNTP and 0.2 μM of each primer under the following conditions: initial denaturation at 95°C for 5 min, followed by 35 cycles of 95°C for 30 s, 53°C for 30 s and 72°C for 1 min, and a final elongation step at 72°C for 6 min. (b) PCR2: the three PCR1 replicates of each sample were then pooled and sent to the GenoToul platform (GeT‐BioPuce, INSA, Toulouse, France). Amplicons were first purified and dosed. Then they were used as templates for the PCR2 to which are added Illumina‐tailed primers targeting the half of Illumina adapters P5 and P7 used in the first PCR and a unique index per sample. After purification, all amplicons were pooled in equimolar concentrations to be sequenced on an Illumina MiSeq system using paired‐end sequencing with a V3 kit (300 bp × 2).

### Metabarcoding analysis

A total of 16,817,628 raw reads across 109 samples, averaging 150,157 reads per sample, were analysed using the DADA2 pipeline (Callahan et al., 2016) in an R 4.2.1 statistical environment (R Core Team, 2020). Sequences were truncated to 250 bp for forward reads and to 240 bp for reverse reads based on the average quality scores. Additionally, reads displaying ‘N’, a quality score below 2, and/or more than 2 expected errors were discarded. The error model was trained using 1,000,000 sequences before denoising, and chimeric sequences were removed based on a consensus approach before the paired ends were assembled. Contaminants were removed using blank controls with the MicroDecon R package (McKnight et al., 2019b).

The final data set contained 10,635,660 reads, with an average of 94,961 sequences per sample after quality filtering. Representative sequences were classified into taxonomic groups using the SILVA 138 database (Quast et al., 2013). Additional filtering on abundance was conducted at a threshold of 0.01% (Bokulich et al., 2013) to remove sequences containing non‐biologically relevant amplicon sequence variants (ASVs) (Breusing et al., 2022). ASVs affiliated with mitochondria sequences of alvinocaridid shrimps were also manually removed from the data set.

Visualisation and statistical analyses of 16S rRNA bacterial diversity were performed using the Phyloseq (v. 1.4.0) (McMurdie & Holmes, 2013) and vegan (v. 2.6.2) (Oksanen et al., 2008) R packages. Alpha diversity across the 109 samples was explored with ASV number for richness and Inverse Simpson Index for evenness. Differences in richness and evenness among categories (hosting organs, vent fields, alvinocaridid host species) were compared with Kruskal–Wallis tests followed by Dunn post‐hoc tests. For Beta diversity, the dataset was normalised via proportions (McKnight et al., 2019a) with the ‘transform_sample_counts’ function (Phyloseq R package) and analysed using Bray–Curtis distance matrices with the ‘distance’ function (Phyloseq R package). Homogeneity between categories was tested with the ‘betadisper’ function (vegan R package), and significant differences between categories were tested by permutational analysis of variance (PERMANOVA; 999 permutations) with the ‘adonis2’ function (vegan R package). Constrained ordinations with stable isotopes ratios for each hosting organs were achieved by canonical analyses on the principal coordinates (CAP) using the ‘ordinate’ (Phyloseq R package) and ‘scores’ (vegan R package) functions.

## RESULTS

### Scanning electron microscopy observations

**FIGURE 2:**
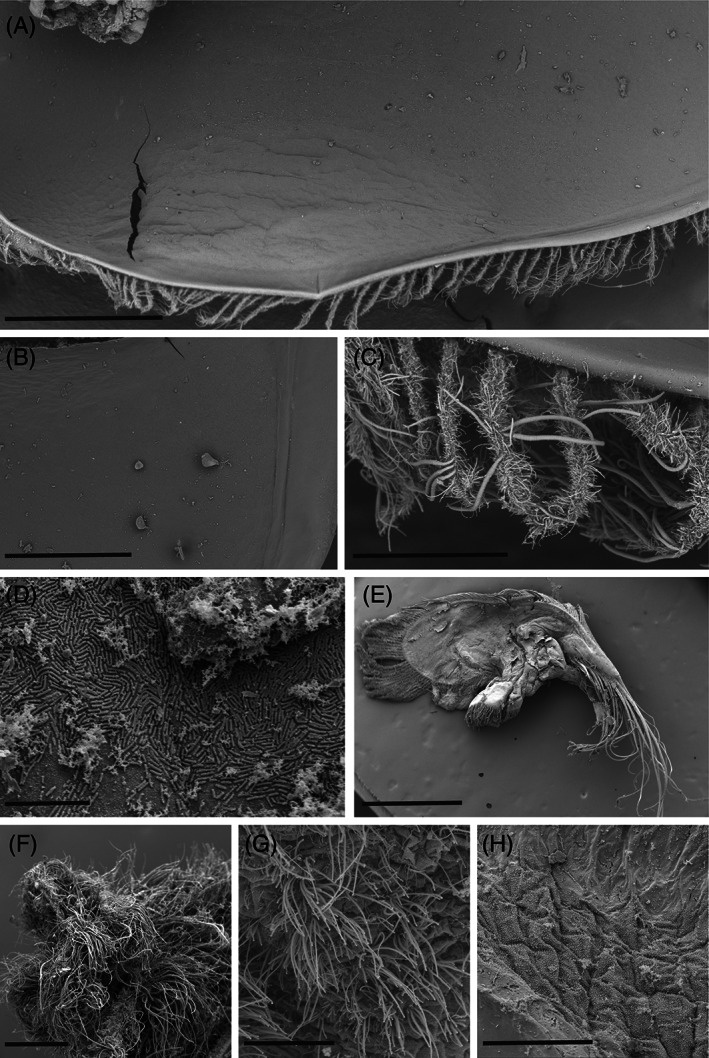
Scanning Electron Microscopy (SEM) observations of microbial communities on the surface of *Rimicaris variabilis* branchiostegites and mouthparts. (A) Overview of the branchiostegite inner side. scale = 1 mm. (B) Enlargement of the branchiostegite inner side devoid of bacterial colonisation; scale = 500 μm. (C) Filamentous bacteria colonising the ventral setae along the external side of the branchiostegite. scale = 100 μm. (D) Single‐layered bacterial mats colonising inner side of *R. variabilis* branchiostegite. scale = 10 μm. (E) Overview of a scaphognathite dorsal side. scale = 1.5 mm. (F) Dense aggregations of filamentous bacteria covering plumose setae of the scaphognathite margin. scale = 200 μm. (G) Filamentous bacteria colonising the scaphognathite surface. scale = 50 μm. (H) Small cocci and rod‐shaped bacteria colonising the scaphognathite surface. scale = 50 μm.

Observation of *Rimicaris variabilis* branchiostegites under Scanning Electron Microscopy (SEM) showed that the inner part of their cephalothoracic cavities was mostly devoid of bacterial colonisation (Figure 2A,B). Conversely, a more abundant bacterial colonisation was observed on the external surface of *R. variabilis* cephalothorax, in particular on setae aligned along their ventral side, which were covered by thick and thin filamentous bacteria (Figure 2C). In 7 out of the 12 *R. variabilis* individuals observed, single‐layered mats of rod‐shaped bacteria were found on their cephalothorax inner surfaces, either on the anterior part facing mouthparts or on the posterior part facing the gills (Figure 2D). In some instances (2 out of 12 individuals), small spots of filamentous bacteria were localised on the most anterior part of the branchiostegites.

Bacterial colonisation was more widespread on *R. variabilis* mouthparts, although remaining limited to particular areas (Figure 2E). Dense aggregations of thick and thin filamentous bacteria‐covered plumose setae distributed along scaphognathite and exopodite margins (Figure 2F). The dorsal and ventral surfaces of these two mouthparts lacked bacteriophore setae (Figure 2E) and were generally only colonised by small cocci and rod‐shaped bacteria on most of their surface (Figure 2G). In 2 out of 10 *R. variabilis* mouthparts observed, ventral and dorsal surfaces of scaphognathites were colonised by filamentous bacteria, but to a lesser extent than marginal setae (Figure 2H).

**FIGURE 3:**
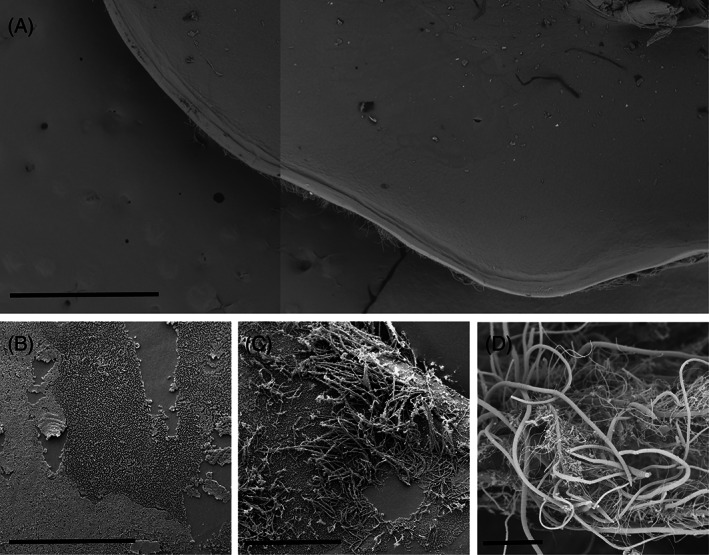
Scanning Electron Microscopy (SEM) observations of microbial communities on the surface of *Nautilocaris saintlaurentae* and *Manuscaris* sp. branchiostegites and mouthparts. (A) Overview (composite image) of the inner side of *N. saintlaurentae* branchiostegite. scale = 1 mm. (B) Single‐layered bacterial mats colonising the inner side of *Manuscaris* sp. branchiostegite. scale = 50 μm. (C) Spot of filamentous bacteria colonising the most anterior part of the *Manuscaris* sp. branchiostegite. scale = 50 μm. (D) Dense aggregations of filamentous bacteria covering plumose setae of *N. saintlaurentae* scaphognathite margin. scale = 50 μm.

Observations of *Nautilocaris saintlaurentae* and *Manuscaris* sp. branchiostegites under SEM revealed similar patterns of bacterial colonisation compared to *R. variabilis* with most of the inner parts of their cephalothoracic cavities devoid of bacteria (Figure 3A) or covered by single layered mats of rod‐shaped bacteria (Figure 3B). As for *R. variabilis*, a few regionalized spots of filamentous bacteria were also present on the most anterior part of the *Manuscaris* sp. branchiostegite, close to the cephalothorax opening (Figure 3C). Bacterial colonisation on mouthparts of these two species was also mostly limited to marginal setae covered by filamentous bacteria (Figure 3D), with only mono layers of cocci and rod‐shaped bacteria on the surfaces of their scaphognathites and exopodites.

### Stable isotopes analysis

*Rimicaris variabilis* and *Nautilocaris saintlaurentae* populations showed limited variations in δ^13^C among vent fields (Kruskal–Wallis, *p* < 0.001; Figure S1A), with only significantly lower δ^13^C values for *R. variabilis* from Susu Knolls compared to those from Tow Cam, Pacmanus and La Scala (Dunn tests, p < 0.001; see Table S2 for detailed *p* values). Slight variations in δ^15^N among vent fields could be observed as well (Kruskal–Wallis, *p* < 0.001; Figure S1B) with higher δ^15^N values in *R. variabilis* from Pacmanus and La Scala compared to those from ABE, Fatu Kapa and Susu Knolls (Dunn tests, *p* < 0.001). Significant differences in δ^34^S of *Rimicaris variabilis* were also found among vent fields (Kruskal–Wallis, *p* < 0.001; Figure S1C) with a trend of ^34^S‐depletion in shrimps from vent fields of the most eastern basins—Manus and Woodlark—compared to shrimp populations from more western basins—North Fiji and Lau—(Dunn tests, *p* < 0.001). At Fatu Kapa, δ^13^C, δ^15^N and δ^34^S of *R. variabilis* and *N. saintlaurentae* were similar between the two species (Dunn tests, *p* > 0.001).

**FIGURE 4:**
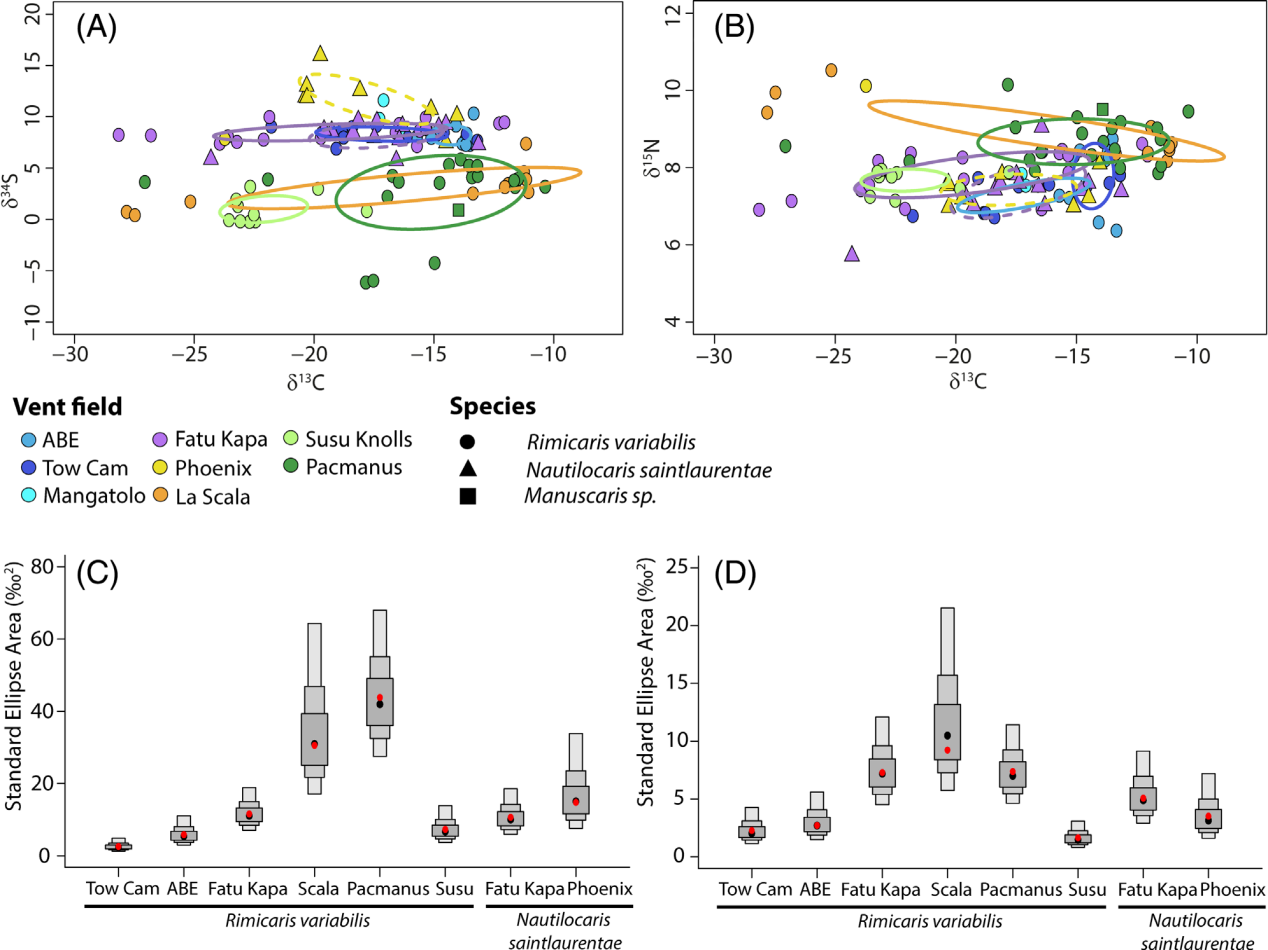
Isotopic niches of alvinocaridid shrimps from southwest Pacific basins. (A) Carbon and sulphur isotopic niches. (B) Carbon and nitrogen isotopic niches. (A,B) Each dot corresponds to the isotopic ratios of a shrimp individual; colours depict hydrothermal vent field locations and shapes depict different alvinocaridid species. (C) Model‐estimated bivariate standard area (SEA_B_) for carbon and sulphur ellipses. (D) Model‐estimated bivariate standard area (SEA_B_) for carbon and sulphur ellipses. (C,D) Boxes in dark grey, medium grey and light grey correspond, respectively, to the 50%, 75% and 95% credibility intervals of probability density function distributions of the model solutions, and black dots are the modes of these distributions. Red dots are the standard ellipse areas computed using a frequentist algorithm adapted for small sample sizes (SEA_C_).

SIBER analysis confirmed that carbon and sulphur isotopic niches of alvinocaridids from Manus and Woodlark basins were clearly separated from those of North Fiji and Lau populations (Figure 4A). However, the same trend was not observed for carbon and nitrogen isotopic niches (Figure 4B) with some overlap between *R. variabilis* from Pacmanus and Fatu Kapa (1.19‰^2^, i.e., 15.3% of the smallest ellipse area), from Tow Cam and Pacmanus (0.74‰^2^, i.e., 28.9% of the smallest ellipse area) or from Tow Cam and La Scala (0.31‰^2^, i.e., 12.1% of the smallest ellipse area). In general, the limited overlap was observed between *R. variabilis* ellipses from vent fields within the same basin with no overlap between the two Manus vent fields and small overlaps between vent fields from the Lau basin (22.4% of the smallest ellipse areas at most), except for carbon and sulphur ellipses of ABE and Fatu Kapa which were strongly overlapping (4.55‰^2^, i.e., 69.12% of the smallest ellipse area). Between Phoenix and Fatu Kapa, carbon and sulphur ellipses of *N. saintlaurentae* overlapped only by 0.64‰^2^ (i.e., 6.5% of the smallest ellipse area) but strongly overlapped for carbon and nitrogen ones (3.45 ‰^2^, i.e., 62.4% of the smallest ellipse area). At Fatu Kapa, ellipses of *R. variabilis* and *N. saintlaurentae* overlapped clearly, in particular for carbon and sulphur ellipses (6.7‰^2^, i.e., 57.7% of the smallest ellipse area) but also for carbon and nitrogen ellipses (2.02‰^2^, i.e., 36.6% of the smallest ellipse area).

Areas of the standard ellipses associated with each shrimp species and vent field populations varied widely (Figure 4C,D), with SEA_c_ values ranging from 1.85‰^2^ (carbon and nitrogen ellipse of *R. variabilis* from Susu Knolls) to 46.24‰^2^ (carbon and sulphur ellipse of *R. variabilis* from Pacmanus). Overall, *R. variabilis* from Pacmanus had the widest isotopic niches (Figure 4C,D), with larger niches than any other shrimp population in nearly all model solutions (>99.99% of model solutions for both carbon and sulphur niches and carbon and nitrogen niches) except for carbon and sulphur niches of *R. variabilis* from La Scala (only 72.09% of model solutions). These broad isotopic niches in some vent fields seemed to result in part from spatial variations, with in general, more similar and clustered isotopic ratios in individuals collected from the same sampling point, particularly at La Scala (Figure S2C). Differences in niche sizes between alvinocaridid species at Fatu Kapa were not well supported by the model, with larger carbon and nitrogen niches for *R. variabilis* in 82.39% of model solutions and larger carbon and sulphur niches in 59.06% of model solutions.

### 16S rRNA metabarcoding analysis

Read distributions among samples following processing and filtering steps varied from 27,759 reads (FU3‐Cr9‐Sc) to 153,987 reads (CHUB‐Cr53‐Sc) resulting in a maximum of 5.5‐fold difference among samples. Alpha Diversity analyses revealed slight variations in ASV richness among host organs (Kruskal–Wallis, *H* = 14.28, *p* < 0.001) or among alvinocaridid species (Kruskal–Wallis, *H* = 6.84, *p* < 0.05) with a significantly higher number of ASVs in stomach compared to mouthparts communities (Dunn's Multiple Comparison Test, *p* < 0.01) and a slightly higher number of ASVs in *Rimicaris variabilis* compared to *Nautilocaris saintlaurentae* communities (Dunn's Multiple Comparison Test, *p* < 0.05). In contrast, ASV richness was similar among back‐arc basins (Kruskal–Wallis, *H* = 7.08, *p* > 0.05) or among vent fields (Kruskal–Wallis, *H* = 7.58, *p* > 0.05). Similarly, Inverse Simpson values did not indicate any variations of evenness among organs, shrimp species, regions or vent fields (Kruskal–Wallis tests, *p* > 0.05).

Based on PERMANOVA analyses, bacterial community composition was significantly influenced mostly by geography (i.e., among vent field; *F* = 4.76, *R* ^*2*^ = 0.224, *p* < 0.001) but also by hosting organs (*F* = 5.94, *R* ^*2*^ = 0.079, *p* < 0.001) or host species (*F* = 3.22, *R* ^*2*^ = 0.043, *p* < 0.001). However, homogeneity of variances among vent fields (betadisper; *F* = 3.349, *p* < 0.01) or shrimp species (betadisper: *F* = 16.409, *p* < 0.001) was not met. Moreover, *R. variabilis* and *N. saintlaurentae* communities composition from Fatu Kapa and Phoenix taken alone—a balanced dataset (betadisper: *F* = 0.483, *p* > 0.05)—did not significantly differ between the two species (PERMANOVA: *F* = 1.742, *R* ^*2*^ = 0.052, *p* > 0.05).

**FIGURE 5:**
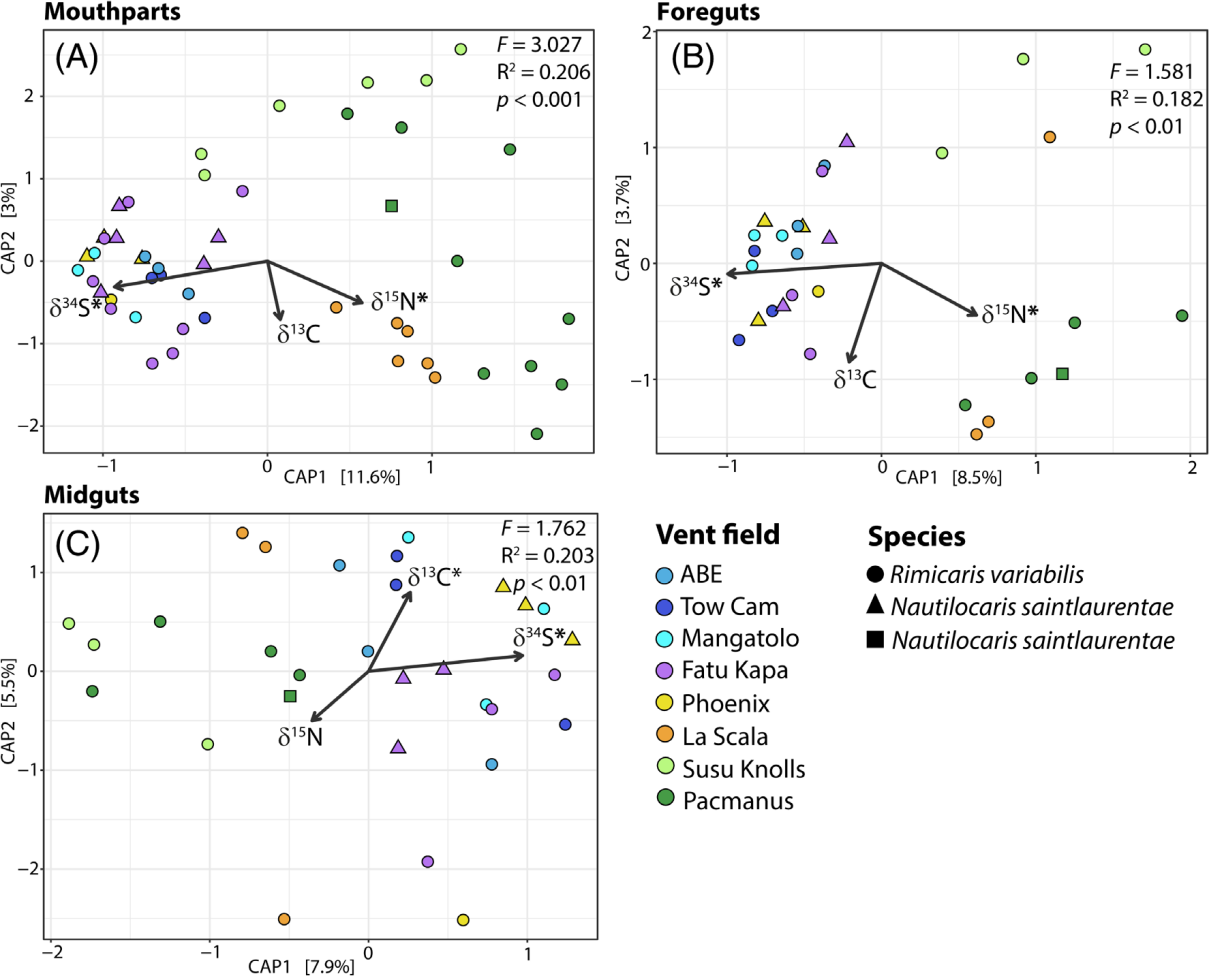
Constrained ordinations of 16S rRNA bacterial diversity by stable isotope ratios using canonical analysis on the principal coordinates (CAP) for each hosting organs. (A) Mouthparts bacterial communities; (B) Foreguts bacterial communities; (C) Midguts bacterial communities. Results from ANOVA‐like permutation tests for CAP are displayed on each plot panel. Stable isotopic ratios that significantly contributed to CAP results are marked with an asterisk (*p* < 0.01, see Table S3). Points are coloured by hydrothermal vent field locations with shapes depicting distinct alvinocaridid species.

Canonical analysis on the principal coordinates (CAP) supported a correlation between stable isotopic composition of abdominal muscles and bacterial communities of alvinocaridid mouthparts (ANOVA‐like: *F* = 3.027, *R* ^*2*^ = 0.206, *p* < 0.001; Figure 5A) with a significant contribution of δ^15^N (*F* = 3.025, *p* < 0.01) and δ^34^S (*F* = 4.556, *p* < 0.001), but not δ^13^C (*F* = 1.5002, *p* > 0.05). RDA models showed similar results for bacterial communities of alvinocaridid foreguts (ANOVA‐like: *F* = 1.581, *R* ^*2*^ = 0.182, *p* < 0.01; Figure 5A) and alvinocaridid midguts (ANOVA‐like: *F* = 1.762, *R* ^*2*^ = 0.203, *p* < 0.001; Figure 5C) with a significant contribution for δ^34^S but not or only slightly for δ^13^C on midguts and not for δ^15^N (see Table S3). PERMANOVA analyses further confirmed that community compositions of each organ were mostly influenced by δ^34^S variations with only a significant influence of δ^15^N for bacterial communities of mouthparts and a slight effect of δ^13^C for bacterial communities of midguts (PERMANOVA tests; see Table S4 for detailed values).

**FIGURE 6:**
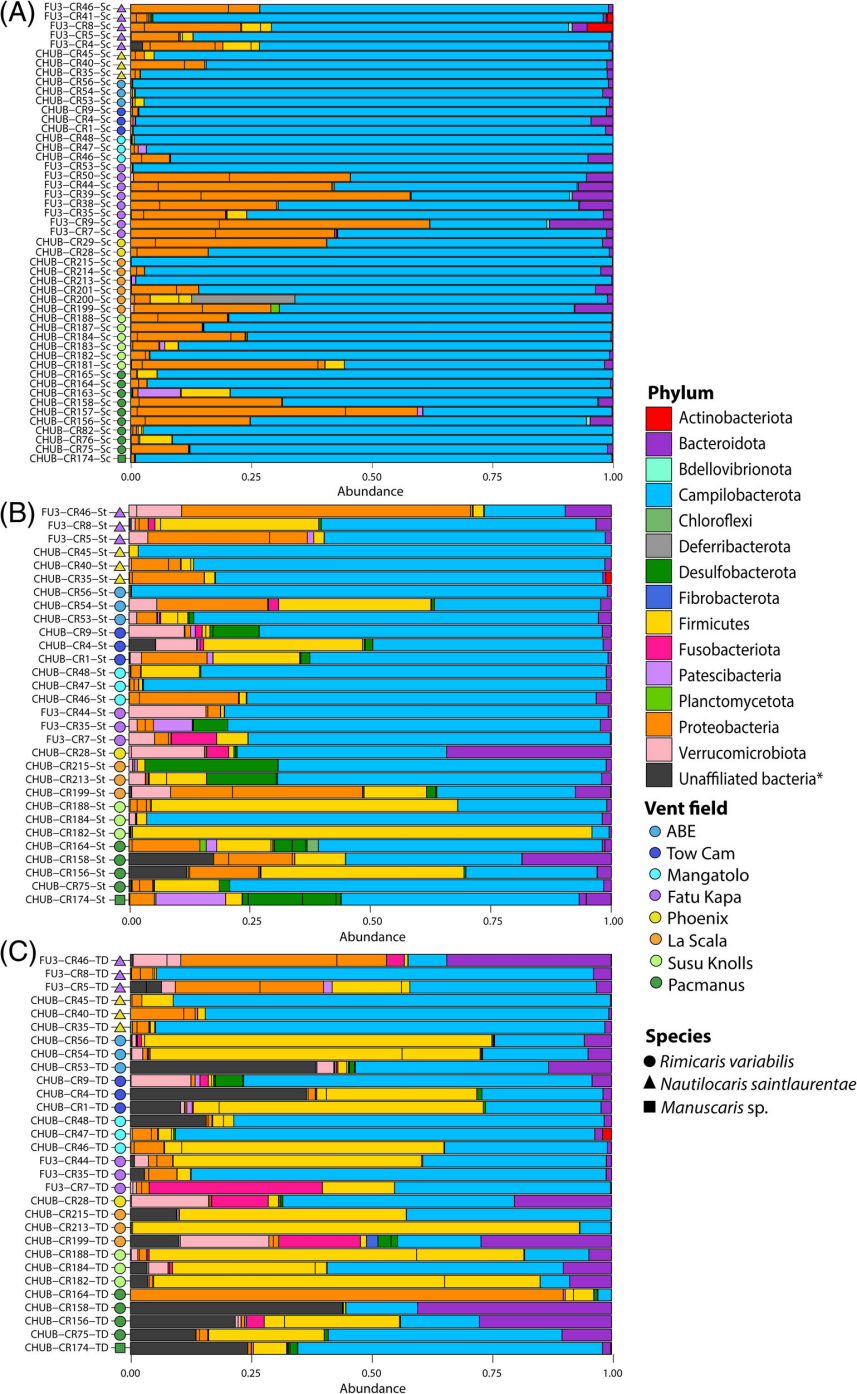
Relative abundances of 16S rRNA gene sequence reads from bacterial communities associated with southwest Pacific alvinocaridids according to their classification at the phylum level (Silva 138 database). *Bacteria unaffiliated at the phylum level according to the Silva 138 database include also some ASVs belonging to the *Deferribacterota* phylum (see Figure 7). (A) Mouthparts bacterial communities; (B) Foreguts bacterial communities; (C) Midguts bacterial communities.

The composition of microbial communities on mouthparts was largely dominated by *Campylobacterota* ASVs (80.5% of mean relative abundance) followed by *Proteobacteria* ASVs (15.4%) (Figure 6A). They also included lower proportions of *Bacteroidota* ASVs (1.9%) and *Firmicutes* ASVs (1.1%); (Figure 6A). *Campylobacterota* ASVs also dominated microbial communities of foreguts (66.4%) and midguts (43.1%), but other groups had much higher relative abundances than on mouthparts, in particular, *Firmicutes* (11.7% in foreguts and 27.6% in midguts, respectively) but also *Verrucomicrobiota* (3.2% in foreguts and 2.9% in midguts respectively) and *Bacteroidota* to a lower extent (3.9% in foreguts and 7.7% in midguts respectively) (Figure 6B,C). In contrast, lower relative abundances of *Proteobacteria* were retrieved both in foreguts (8.5%) and in midguts (7.4%). Substantial proportions of *Desulfobacterota* (2.8%) and *Patescibacteria* (1.1%) were also found in foreguts; (Figure 6B) and of *Fusobacteriota* in midguts (2.5%); (Figure 6C). Large proportions of bacterial sequences, not affiliated to any particular phylum according to the Silva 138 database, were observed as well in foreguts (1.1%) and in midguts (7.7%).

**FIGURE 7:**
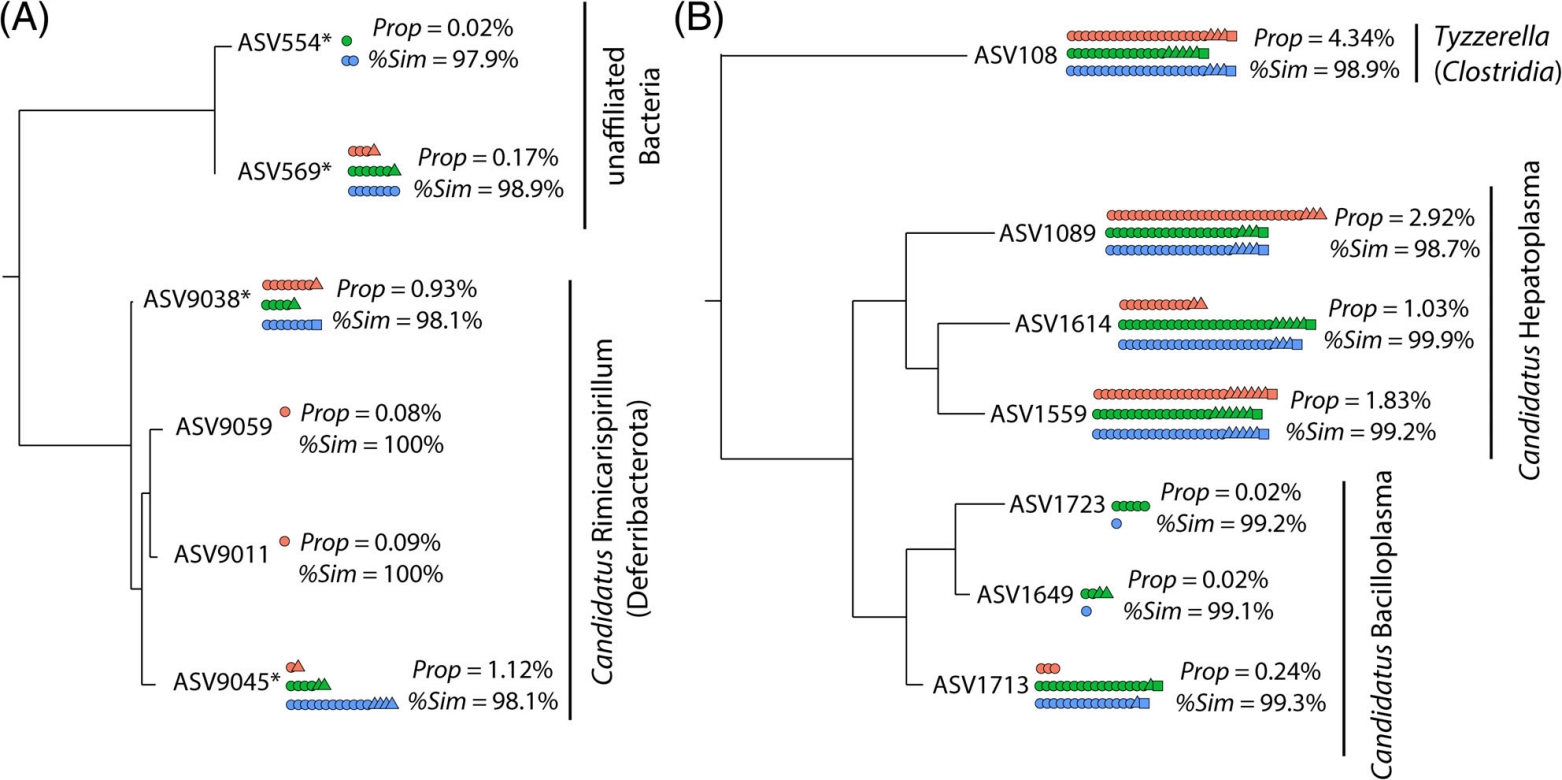
Phylogenetic trees of (A) *Deferribacterota* and unaffiliated bacterial ASVs and (B) *Firmicutes* ASVs agglomerated by similarity (*h* = 0.1). Panel A: Bacterial ASVs without phylum affiliation with the Silva 138 database are marked with an asterisk. All panels: Trees were constructed with the Maximum Likelihood method, based on the General Time Reversible model with Gamma distribution and allowing for some sites to be invariable (GTR + I + G). Prop: Relative abundance of the ASV among total sequence reads within the dataset. %Sim: % of similarity with the ASV best BLAST hit (*R. exoculata* or *R. chacei* digestive epibiont; details on Table S5). Each dot represents the occurrence of the lineage in an individual with shapes depicting alvinocaridid species (circle: *R. variabilis*; triangle: *N. saintlaurentae*; square: *Manuscaris* sp.) and colours depicting the hosting organ (red: mouthpart; green: stomach; blue: digestive tube).

Best BLAST hits of these unaffiliated ASVs with the NCBI database revealed two main bacterial lineages, the most abundant lineage showing clear sequence similarity with *R. chacei* and *R. exoculata Deferribacterota* gut symbionts (98.1% for both ASVs) whereas the other corresponded to an unknown bacterial lineage similar to a symbiont retrieved from a cuttlefish nidamental gland (97.9% and 98%); (Figure 7A and Table S5). Phylogenetic reconstruction of these unaffiliated ASVs agglomerated by phylogenetic similarity together with ASVs affiliated to *Deferribacterota* (ASV9011 and ASV9059) confirmed their similarity with the ‘unaffiliated’ *Deferribacterota* ASVs (ASV9038 and ASV9045) (Figure 7A). The *Deferribacterota* ASV9011 and ASV9059 were identical to a gut symbiont of *R. chacei* (Table S5) but were only found in the mouthpart of one *R. variabilis* individual (CHUB‐Cr200‐Sc). On the other, the *Deferribacterota* ASV9038 and ASV9045 were retrieved within each hosting organ and each alvinocaridid species (Figure 7A), although these were notably absent from all organs of seven individuals (CHUB‐Cr28, CHUB‐Cr40, CHUB‐Cr45, CHUB‐Cr47, CHUB‐Cr164, FU3‐Cr7 and FU3‐Cr44).

Phylogenetic reconstruction of *Firmicutes* ASVs agglomerated by phylogenetic similarity showed three main bacterial lineages in this phylum (Figure 7B) with three ASVs affiliated to the *Candidatus* Hepatoplasmata genus (class Bacilli), three ASVs affiliated to the *Candidatus* Bacilloplasma genus (class Bacilli) and one ASV affiliated to the *Tyzzerella* genus (class *Clostridia*). Best BLAST hits of these ASVs were always *Rimicaris exoculata* or *Rimicaris chacei* foreguts and midguts symbionts with sequence similarity comprised between 98.7% and 99.9% (Figure 7B and Table S5). Most *Firmicutes* ASVs were present within each hosting organs and among each alvinocaridid species except ASV1723 that was found within *R. variabilis* foreguts and midguts only and ASV1649 that was only within *R. variabilis* and *N. saintlaurentae* foreguts and midguts (Figure 7B).

## DISCUSSION

### Nutritional strategies of alvinocaridid shrimps from hydrothermal vents of the Southwest Pacific basins

Our observations on the inner side of the cephalothoracic cavities showed only a scarce coverage of bacterial epibionts for either *Rimicaris variabilis*, *Nautilocaris saintlaurentae* or *Manuscaris* shrimps (Figures 2 and 3). A slightly more developed colonisation was observed on their mouthparts with some filamentous bacteria although limited to particular areas, mostly the plumose setae on the mouthpart margins. This sharply contrasts with colonisation patterns seen not only in alvinocaridid species relying mostly on their cephalothoracic chemosymbiosis such as *Rimicaris exoculata* (Petersen et al., 2010; Zbinden et al., 2004; Zbinden & Cambon‐Bonavita, 2020) or *Rimicaris kairei* (Methou et al., 2022b), but also in those with a mixed diet, only partially dependent on this symbiosis like *Rimicaris chacei* (Apremont et al., 2018), and which all exhibit extensive colonisation of their cephalothoracic cavities by filamentous bacteria. Although earlier works stress out the importance of the moult cycle in *R. exoculata* symbiosis (Corbari et al., 2008), with a sparse colonisation within the cephalothoracic cavity in early moult stage individuals, it is unlikely that moult stages have introduced a bias in our observations for alvinocaridids from South West Pacific basins. Indeed, the inversely dense colonisation of ventral setae along the external face of their cephalothorax (Figure 2C) suggests that limited colonisation of their branchiostegites does not stem from a recent renewal of the exoskeleton but is found all along their moult cycle.

In addition, no clear correlation between the relative abundance of epibionts colonising their mouthparts and stable isotopes ratios of carbon could be established among alvinocaridid individuals analysed in our study (Figure 5). In hydrothermal vent ecosystems, these variations in δ^13^C are mostly attributed to the use of different carbon fixation pathways by chemosynthetic microorganisms, with depleted δ^13^C ratios for those using the CBB cycle (typically −36%–−30‰) and enriched δ^13^C ratios for rTCA‐fixed carbon sources (typically −15%–−10‰) (Hügler & Sievert, 2011; Portail et al., 2018). Both carbon fixation pathways can be found in chemosynthetic epibiont communities with *Campylobacterota* using the rTCA cycle and *Proteobacteria* using the CBB cycle (Cambon‐Bonavita et al., 2021; Jan et al., 2014; Jiang et al., 2020). However, the relationship between individual δ^13^C ratios and the relative abundance of rTCA‐ or CBB‐fixing bacterial lineages did not hold for our dataset. As an example, the *R. variabilis* individual exhibiting the highest relative abundance of *Proteobacteria* lineages within its mouthparts (FU3‐CR9; see Figure 5.) had a more enriched δ^13^C ratio (−16.6‰) compared to a *R. variabilis* individual from the same site (FU3‐CR53; −23.6‰) whose mouthparts were completely dominated by *Campylobacterota* lineages and *Proteobacteria* being almost absent. Collectively, these results from microscopic observations, bacterial diversity, and isotopic ratios all suggest that neither *R. variabilis*, *N. saintlaurentae* nor *Manuscaris* sp. rely on chemosynthetic epibionts as their main source of nutrition.

Aside from a chemosymbiotic diet, other feeding modes such as bacterial grazers, scavengers or detritivores have been proposed for alvinocaridids shrimps, including those from the Manus and North Fiji basins (Suh et al., 2022; Van Audenhaege et al., 2019). Our results are consistent with previous studies on *R. variabilis* (Suh et al., 2022; Van Audenhaege et al., 2019), showing large trophic niches with particularly variable isotopic composition for carbon. Still, with a maximum of 11.6‰ for δ^34^S, their feeding sources remain within the range of chemosynthetically derived organic matter with no clear input of photosynthetic origin (Erickson et al., 2009; Reid et al., 2013; Van Dover & Fry, 1994). A notable exception was the *N. saintlaurentae* sampled at the Phoenix site (North Fiji basin), with a clearly higher δ^34^S ratio, up to 15.6‰, pointing out a potential mixed diet for this species with a larger contribution of photosynthetic material. However, the small size of these individuals and the observation of red lipid storages during their dissection (Methou, personal observation) rather indicate the influence of an ontogenetic shift as seen in juveniles and subadults stages of alvinocaridid shrimps from the Mid Atlantic Ridge (Methou et al., 2020; Pond et al., 1997), Central Indian Ridge (Van Dover, 2002) or the Mariana Arc (Stevens et al., 2008). Overall, these results suggest a generalist behaviour with various potential chemosynthetic food sources at the species level but more specialised feeding habits at a local scale. This is supported by the more similar and clustered isotopic ratios of shrimp individuals from the same sampling point within a vent field, arguing for a relatively strong habitat fidelity (Figure S2A–D). Thus, although being potentially highly mobile animals, alvinocaridids from the southwest Pacific might remain faithful to the same assemblage of foundation species once they settled, or at least at the timescales integrated by stable isotopic compositions of their abdominal muscles. Nevertheless, the presence of large alvinocaridid assemblages on chimney outcrops (Figure 1A), outside of mussel beds, tubeworm bushes or hairy snail colonies, indicates that these shrimps do not solely rely on detritus of these foundational symbiotrophs but are also able to feed on other nutrition sources, such as bacterial mats, possibly. Thereby, both detritivory and bacterivory diets could coexist in *R. variabilis* and *N. saintlaurentae*, although with strong intraspecific variations among individuals.

Interestingly, no isotopic niche partitioning was observed between *R. variabilis* and *N. saintlaurentae* from Fatu Kapa suggesting similar diets for the two co‐occurring species (Figure 4). Since both were sometimes collected from the same assemblage, there was no clear indication of spatial segregation in distinct habitat either. To avoid competitive exclusion, niche theory predicts that sympatric species differ by their resource use and/or spatio‐temporal habitat distribution, particularly in the case of closely related species with similar morphological traits and/or limited resource availability (Hutchinson, 1957; Schoener, 1974). However, the high biological productivity of hydrothermal vent ecosystems coupled with their temporal instability at short time scales might not allow to overreach the carrying capacity of these environments on the resources used by these shrimps, enabling long‐term coexistence of similar species for the same food source. This contrasts with *Rimicaris* shrimps co‐occurring in high densities assemblages on the Mid Atlantic Ridge, for which clear spatial and trophic niche partitioning could be observed between *R. exoculata* and *R. chacei* (Methou et al., 2020; Methou et al., 2022a). In the case of these species relying on their chemosymbiosis, competition for food is interlinked with competition for a limited space—that is, the access to the vent fluid source—resulting ultimately in niche partitioning for the case of vent holobionts (Beinart et al., 2012; Methou et al., 2022a; Van Audenhaege et al., 2019). On the other hand, vent species with a distinct type of diet, such as alvinocaridids from the southwest Pacific might experience a more relaxed competition enabling co‐occurring species to occupy the same ecological niche.

### Resident bacterial communities within the digestive system of alvinocaridid shrimps

Although bacterial coverage on mouthparts of *R. variabilis*, *N. saintlaurentae* and *Manuscaris* sp. was very low comparatively to *Rimicaris* species from the Atlantic or Indian Oceans, the composition of their epibiotic communities mirrors those previously observed in cephalothoracic cavities of the latter (Apremont et al., 2018; Cambon‐Bonavita et al., 2021; Guri et al., 2012; Jan et al., 2014; Methou et al., 2022b; Petersen et al., 2010; Zbinden et al., 2008). Thus, we found a similar phylogenetic diversity with a dominance of *Campylobacterota*, followed by several families of *Proteobacteria*—including *α‐*, *γ‐* and *ζ‐proteobacteria*—as well as *Bacteroidota* epibionts.

In contrast, composition of their digestive communities differs, in part, from those of *R. exoculata* and *R. chacei* in the Mid Atlantic Ridge (Apremont et al., 2018; Aubé et al., 2022; Durand et al., 2010, 2015; Guéganton et al., 2022) or *R. kairei* in the Central Indian Ridge (Qi et al., 2022), which exhibited a clear partitioning of bacterial lineages between their digestive organs. Indeed, bacterial communities of their midguts were mainly composed of Deferribacterota and *Firmicutes* from the *Clostridia* class, whereas *Firmicutes* affiliated to *Mycoplasmatales* (class *Bacilli*) were dominant in foreguts (Aubé et al., 2022; Durand et al., 2010; Guéganton et al., 2022). These two phyla constitute resident communities within their respective hosting organs in *R. exoculata* and *R. chacei*, whereas other bacterial lineages such as *Campylobacterota* or *Gammaproteobacteria* were only observed in the alimentary bolus (Guéganton et al., 2022).

In the three species of alvinocaridids from the southwest Pacific, Deferribacterota affiliated to *Candidatus* Rimicarispirillum and several lineages of *Firmicutes* affiliated to *Mycoplasmatales* and *Clostridia* were found in both the midguts and foreguts communities of every shrimp individual, often constituting the dominant lineages within their community (Figure 5B,C). These *Firmicutes* and *Deferribacterota* were also found on the mouthparts of some individuals but in lower proportions than in their digestive systems (Figure 6A). This absence of partitioning among hosting organs in alvinocaridids from the Southwest Pacific is thus more similar to the case of the terrestrial isopod *Armadillidium vulgare* whose *Firmicutes* symbiont, *Candidatus* Hepatoplasma crinochetorum, occupying predominantly their hepatopancreas and caeca (Bouchon et al., 2016; Wang et al., 2004), was also found in other tissues, including their hindgut, nerve cord, gonads as well as their haemolymph and faeces (Dittmer et al., 2016).

It is surprising that only *Deferribacterota* ASVs identical to known sequences were correctly affiliated and not those with 98.1% similarity based on the Silva 138 database (Figure 7). Recent metagenomics analyses of *R. exoculata* gut symbiont showed low alignment coverages of these *Deferribacterota* symbionts with other known lineages within this phylum leading to the creation of a new genus *Candidatus* Rimicarispirillum within a new family *Candidatus* Microvillispirillaceae for their classification (Aubé et al., 2022). This low alignment coverage could explain their affiliation failure with the current reference database for 16S rRNA metabarcoding studies and highlights may be a need to revise the current classification for this phylum.

Our results also reveal that the closest known lineages of *Deferribacterota* and *Firmicutes* found within the foreguts and midguts of southwest Pacific alvinocaridids were systematically symbionts associated with *R. exoculata* or *R. chacei* (Figure 7.) supporting their vertical inheritance and an association maintained along the evolutionary history of these hydrothermal vent shrimps. More broadly in the case of *Firmicutes*, related lineages like *Candidatus* Hepatoplasma or *Candidatus* Bacilloplasma were also retrieved in digestive systems of several crustaceans such as terrestrial, intertidal or deep‐sea isopods (Bouchon et al., 2016; Dittmer et al., 2016; Eberl, 2010; Fraune & Zimmer, 2008; Wang et al., 2004, 2016), hadal amphipods (Cheng et al., 2019) or coastal crab and shrimp species (Chen et al., 2015; Zhang et al., 2014, 2016). These *Candidatus* Hepatoplasma symbionts exhibited a high level of specificity with their hosts in terrestrial isopods (Fraune & Zimmer, 2008). Taken together, these results would even suggest an ancient and evolutionarily conserved partnership in the crustacean subphylum.

Conversely, the presence of each of these *Deferribacterota* and *Firmicutes* lineages within the digestive system of distantly‐related alvinocaridids like *R. variabilis* and *N. saintlaurentae* but from the same geographic area (Figure 7.) is more congruent with a horizontal mode of transmission. Similarly, hepatopancreas of the co‐occurring intertidal isopods, *Ligia pallasii* and *L. occidentalis*, hosted the same lineage of *Candidatus* Hepatoplasma (Eberl, 2010). It has been suggested that inter‐moults and inter‐generational transmission of *Mycoplasmatales* and *Deferribacteres* symbionts could be achieved by trophallaxis among individuals or by ingestion of their old cuticle (Durand et al., 2015). In the light of our results, this reinfection must be possible not only among individuals from the same species but also among individuals from the same family.

## CONCLUSION

Our study confirms that these opportunistic alvinocarids from the South West Pacific basins do not rely heavily on chemosymbiosis as an alternative or complementary part of their diet. Rather, they most likely feed on other food sources available at vent ecosystems, including bacterial mats, detritus or mucus discarded by the foundational symbiotroph species. On the other hand, their digestive microbiome, notably bacteria from the *Firmicutes* and *Deferribacterota* group, was highly conserved compared to other alvinocaridids but also more largely among crustaceans, suggesting overall a possible ancient and evolutionarily conserved bacterial partnership. However, the distribution of these *Firmicutes* and *Deferribacterota* lineages within the different organs of the digestive system differs from those of other alvinocaridids where they are mostly restricted to the foregut and the midgut respectively. A larger sampling comparing digestive microbiomes of different alvinocaridid species from several regions would be required to disentangle the respective influence of geography, host diet and host phylogeny of these associations.

## AUTHOR CONTRIBUTIONS

**Pierre Methou:** Conceptualization (lead); data curation (lead); formal analysis (lead); investigation (lead); methodology (lead); validation (lead); visualization (lead); writing – original draft (lead); writing – review and editing (equal). **Valérie Cueff‐Gauchard:** Data curation (supporting); formal analysis (equal); methodology (equal); resources (supporting); validation (supporting); visualization (equal); writing – review and editing (equal). **Loïc N Michel:** Data curation (equal); formal analysis (equal); investigation (equal); methodology (equal); resources (equal); validation (equal); writing – review and editing (equal). **Nicolas Gayet:** Investigation (supporting); methodology (equal). **Florence Pradillon:** Data curation (equal); resources (equal); supervision (equal); validation (equal); writing – review and editing (equal). **Marie‐Anne Cambon‐Bonavita:** Funding acquisition (lead); project administration (lead); resources (equal); supervision (lead); validation (equal); writing – review and editing (equal).

## CONFLICT OF INTEREST STATEMENT

The author declares no competing interests.

## Supporting information


**Figure S1.** Isotopic ratios of alvinocaridids from Southwest Pacific basins for (A) carbon, (B) nitrogen and (C) sulphur.
**Figure S2.** Carbon and sulphur isotopic ratios of alvinocaridid shrimps highlighting sampling events at (A) the Pacmanus field; (B) Susu Knolls field; (C) La Scala field; and (D) Fatu Kapa field.

**Table S1.** Summary of alvinocaridid shrimp sampling and conducted analyses with individual GenBank Identifier.

**Table S2.** *p* values of Dunn tests used for isotopic ratio comparisons (δ^13^C, δ^15^N δ^34^S) among alvinocaridid species and vent fields.

**Table S3.** Results from ANOVA‐like permutation tests for RDA by term with 999 permutations for each hosting organs.

**Table S4.** Results from PERMANOVA tests of isotopic ratios with 999 permutations for each hosting organs.

**Table S5.** Best Blast hit results of Firmicutes ASVs from hosting organs of SW Pacific alvinocaridid shrimps

## Data Availability

All COI barcode sequences have been deposited in GenBank under accession numbers OQ363903 – OQ364004 (see Table S1 for sampling summary with associated individual ID). The 16S rRNA dataset is available in the NCBI SRA repository (submission identifier SUB12697284 and BioProject identifier PRJNA932596). All stable isotopes measurements are available in the DeepIso database (https://www.seanoe.org/data/00654/76595/).
