# Supplementary material for: Symbioses of alvinocaridid shrimps from the South West Pacific: No chemosymbiotic diets but conserved gut microbiomes
Source: Environ Microbiol Rep. 2023 Sep 26;15(6):614–30. doi: 10.1111/1758-2229.13201 (PMC10667644; doi:10.1111/1758-2229.13201)
Supplement: Supplementary file 1 — Figure S1. Isotopic ratios of alvinocaridids from Southwest Pacific basins for (A) carbon, (B) nitrogen and (C) sulphur. Figure S2. Carbon and sulphur isotopic ratios of alvinocaridid shrimps highlighting sampling events at (A) the Pacmanus field; (B) Susu Knolls field; (C) La Scala field; and (D) Fatu Kapa field. [file EMI4-15-614-s002.pdf]

Supplementary material

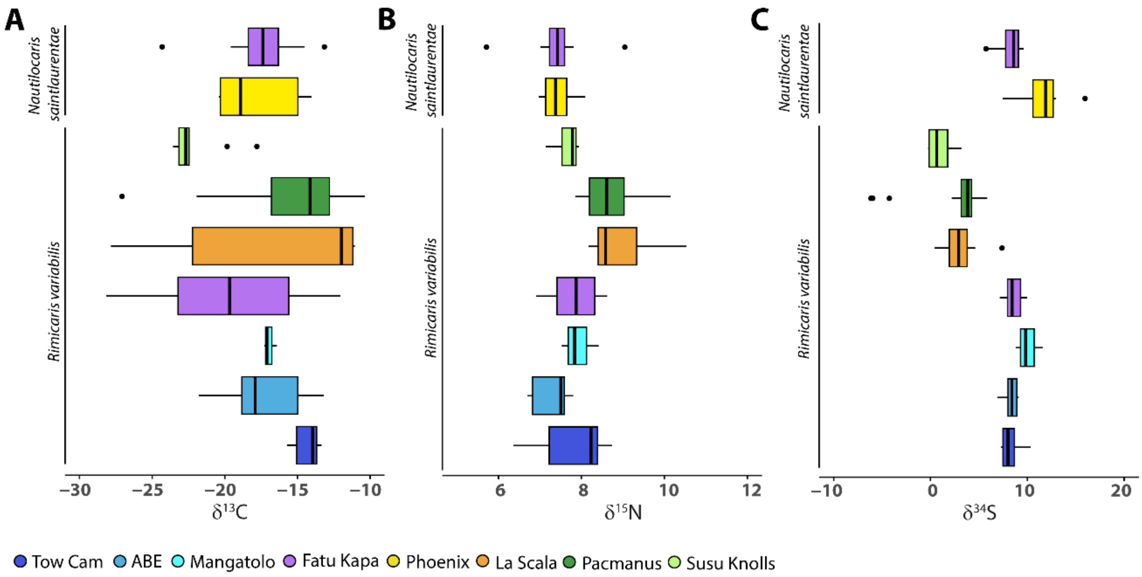

Figure S1. Isotopic ratios of alvinocaridids from Southwest Pacific basins for **A.** Carbon **B.** Nitrogen and **C.** sulfur.

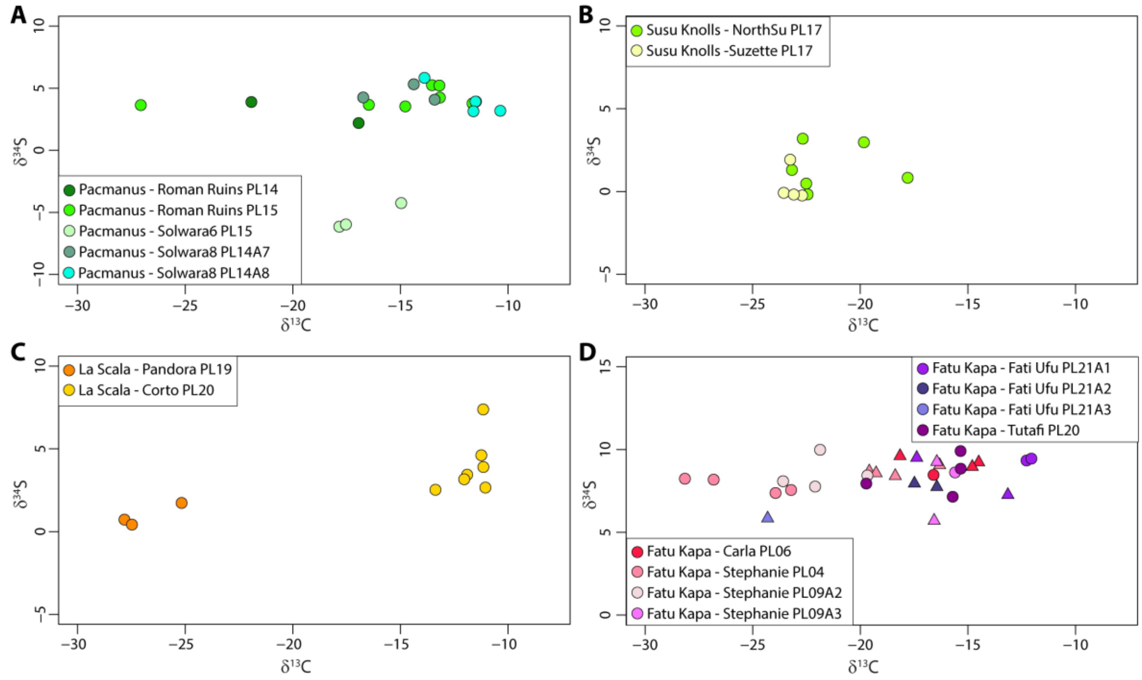

Figure S2. Carbon and sulfur isotopic ratios of alvinocaridid shrimps highlighting sampling events at **A**. the Pacmanus field **B.** Susu Knolls field **C.** La Scala field **D.** Fatu Kapa field.

Table S1. Summary of alvinocaridid shrimp sampling and conducted analyses with individual GenBank Identifier.

Table S2. p-values of Dunn tests used for isotopic ratio comparisons ($\delta^{13}C$, $\delta^{15}N$ $\delta^{34}S$) among alvinocaridid species and vent fields.

Table S3. Results from ANOVA-like permutation tests for RDA by term with 999 permutations for each hosting organs.

Table S4. Results from PERMANOVA tests of isotopic ratios with 999 permutations for each hosting organs.

Table S5. Best Blast hit results of Firmicutes ASVs from hosting organs of SW Pacific alvinocaridid shrimps.
